# Cytokine and Antioxidant Regulation in the Intestine of the Gray Mouse Lemur (*Microcebus murinus*) During Torpor

**DOI:** 10.1016/j.gpb.2015.03.005

**Published:** 2015-06-17

**Authors:** Shannon N. Tessier, Barbara A. Katzenback, Fabien Pifferi, Martine Perret, Kenneth B. Storey

**Affiliations:** 1 Institute of Biochemistry & Department of Biology, Carleton University, Ottawa, ON K1S 5B6, Canada; 2 UMR 7179 Centre National de la Recherche Scientifique, Muséum National d’Histoire Naturelle, Brunoy 91800, France; 3 Department of Surgery & Center for Engineering in Medicine, Massachusetts General Hospital & Harvard Medical School, Charlestown, MA 02129, USA; 4 Department of Biology, University of Waterloo, Waterloo, ON N2L 3G1, Canada

**Keywords:** Primate torpor, Cytokines, Chemokines, Antioxidant enzymes, Gut immunology

## Abstract

During food shortages, the gray mouse lemur (*Microcebus murinus*) of Madagascar experiences daily torpor thereby reducing energy expenditures. The present study aimed to understand the impacts of torpor on the immune system and antioxidant response in the gut of these animals. This interaction may be of critical importance given the trade-off between the energetically costly immune response and the need to defend against pathogen entry during hypometabolism. The protein levels of cytokines and antioxidants were measured in the small intestine (duodenum, jejunum, and ileum) and large intestine of aroused and torpid lemurs. While there was a significant decrease of some pro-inflammatory cytokines (IL-6 and TNF-α) in the duodenum and jejunum during torpor as compared to aroused animals, there was no change in anti-inflammatory cytokines. We observed decreased levels of cytokines (IL-12p70 and M-CSF), and several chemokines (MCP-1 and MIP-2) but an increase in MIP-1α in the jejunum of the torpid animals. In addition, we evaluated antioxidant response by examining the protein levels of antioxidant enzymes and total antioxidant capacity provided by metabolites such as glutathione (and others). Our results indicated that levels of antioxidant enzymes did not change between torpor and aroused states, although antioxidant capacity was significantly higher in the ileum during torpor. These data suggest a suppression of the immune response, likely as an energy conservation measure, and a limited role of antioxidant defenses in supporting torpor in lemur intestine.

## Introduction

A variety of small mammals use torpor to reduce energy expenditures and enhance survival during inactive parts of the day especially when faced with stressful conditions (*e.g.*, nutrient or water limitation, or low ambient temperatures). By suppressing metabolic rate and allowing core body temperature (*T*_b_) to fall, animals extend the time that they can survive using internal fuel reserves alone. The gray mouse lemur (*Microcebus murinus*) uses daily torpor cycles during the dry season as an energy conservation mechanism, which is thought to be related to the extreme fluctuations of the resource availability in the seasonally-arid regions of Madagascar where this species inhabits [1,2]. Indeed, as conditions dictate, these lemurs can also extend torpor into multi-day bouts of hibernation, a capability previously-unknown for primate species. Daily torpor lasts 9.3 h on average but may be as long as 17.5 h [2]. During torpor, *T*_b_ can vary substantially from relatively high values of 28–32 °C to recorded lows of 7.8 °C, with mean values around 17.3 °C (depending in part upon the ambient temperature) [2]. Lemurs exhibit low resting metabolic rates and can further reduce their basal metabolic rate to 20%–30% of their euthermic counterparts by entering torpor, thereby conserving significant amounts of fuel and energy [2–5]. The reallocation of energy resources toward essential metabolic processes may create energy deficits in other “non-essential” physiological processes, such as the activity of immune system.

Mucosal tissues, such as the intestinal mucosa, are primary sites of entry for pathogens. Under normal conditions, the specialized immune cells and molecules that comprise the mucosal immune system defend against pathogen entry and replication, thereby maintaining animal health. However, the immune system is energetically costly to maintain and activate and may be selectively regulated during periods of hypometabolism, thereby compromising mucosal immunity and predisposing animals to disease during this time. For example, modulation of the intestinal immune system during seasonal hibernation has been reported in some small mammals such as ground squirrels [6]. This is thought to occur in part due to energetic constraints under hypometabolic conditions, but may also be linked to atrophy of the intestinal tract villi or a shifting microbial environment resulting from prolonged or repeated fasting over the winter months [7]. A number of soluble mediators regulate the recruitment, activation, and deactivation of innate immune cells. Chemokines such as monocyte chemoattractant protein-1 (MCP-1), macrophage inflammatory protein-1 alpha (MIP-1α), and macrophage inhibitory protein-2 (MIP-2) regulate the recruitment of immune cells [8–10]. In addition, tumor necrosis factor-alpha (TNF-α), macrophage colony-stimulating factor (M-CSF), interferon-gamma (IFN-γ), interleukin-12 subunit p40 (IL-12p40), IL-12p70, IL-1β, and IL-6 regulate the pro-inflammatory processes, whereas IL-10 and transforming growth factor-beta (TGF-β) act as anti-inflammatory mediators [8–10]. Together, these cytokines provide an overview of the regulation of the immune response. The balance between pro-inflammatory and anti-inflammatory cytokines mediates homeostasis in an organism, while the selective regulation and proper functioning of the immune response are important for defense against invading pathogens.

Oxidative stress pathways are intricately linked to immune function and play critical roles in the general health of the gastrointestinal tract. The immune system uses oxidants as a mechanism of killing pathogens. For example, phagocytes produce both reactive oxygen species (ROS) and reactive nitrogen species (RNS), such as superoxide and nitric oxide, which are highly toxic and damaging to target pathogens [11]. While enhanced antioxidant defenses plays a role in deep torpor, antioxidant responses also play crucial roles during a rapid rise in ROS generation associated with the huge increase in oxygen uptake and consumption that powers arousal from torpor [12]. Indeed, the involvement of antioxidant enzymes and metabolites appears to be an essential characteristic of most forms of hypometabolism [13], including playing an established cytoprotective role in the intestine of hibernating mammals [14]. Important antioxidant enzymes include superoxide dismutase (SOD), present as the Cu/Zn-dependent cytoplasmic form (SOD1) and the Mn-dependent mitochondrial form (SOD2), catalase, peroxiredoxin 2 (PRX2), and thioredoxin (TRX1).

The present study examines the regulation of cytokines and antioxidants in the small and large intestine of aroused and torpid gray mouse lemurs. Multiplex and total antioxidant assays were used to determine the relative levels of pro-inflammatory cytokines, anti-inflammatory cytokines, chemokines, and antioxidant enzymes as well as antioxidant metabolites. The differential levels of cytokines in the various intestinal sections of lemurs during torpor suggest regulation of the local immune response in the gut of lemurs during hypometabolism. However, total protein levels of antioxidant enzymes did not change in response to torpor, although minimal changes in total antioxidant capacity (the sum of low molecular mass antioxidants) were observed in the ileum during torpor. These data suggest either low involvement of antioxidant defenses in the torpor response, or that antioxidant defenses are responsive to other factors not identified in the present study.

## Results

### Regulation of pro-inflammatory cytokine protein levels in the intestine during torpor

**Figure 1:**
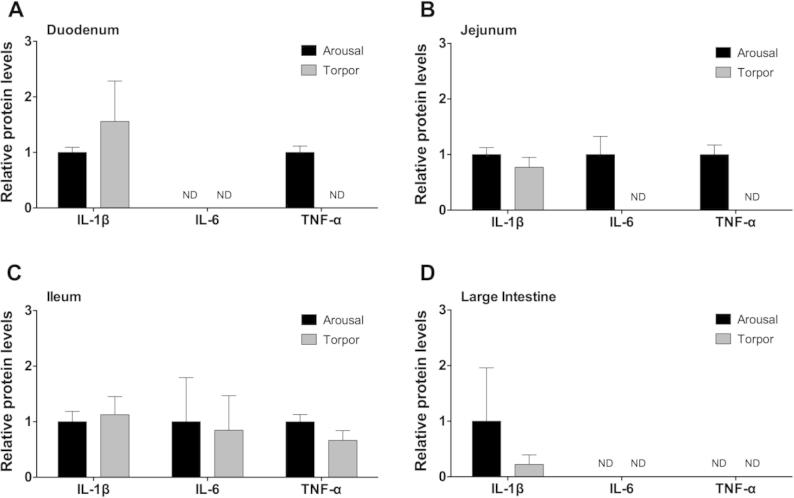
**Relative protein levels of pro-inflammatory cytokines in the intestine of aroused and torpid lemurs** IL-1β, IL-6, and TNF-α protein levels were measured in the duodenum (**A**), jejunum (**B**), ileum (**C**), and large intestine (**D**) using the median fluorescent intensity (MFI) as measured by Luminex®. Data are mean ± SEM (*n* = 3–4) where the values for arousal groups are normalized to a relative value of 1 and torpid groups are expressed as a relative fold change to arousal values. Statistical analysis was performed using a Student’s two-tailed *t*-test, assuming unequal variances. ND, not detected, indicating that cytokine protein levels were below the detection limit of the assay.

To assess the regulation of pro-inflammatory cytokines in the duodenum, jejunum, ileum, and large intestine, the relative protein levels of IL-1β, IL-6, and TNF-α were examined in torpid and aroused animals. While IL-1β was detected in all intestine samples of torpid and aroused animals, no significant differences were observed between torpid and aroused animals (Figure 1). IL-6 protein was detected in the ileum of aroused and torpid lemurs, however, there was no significant difference between these two states (*P* > 0.05, Figure 1C). The levels of IL-6 were below the detection limit of the multiplex kit in the duodenum (Figure 1A for both), jejunum (Figure 1B for torpid animals), and large intestine (Figure 1D for both). Similarly, TNF-α was detected in the ileum of aroused and torpid animals, but without significant difference (Figure 1C). TNF-α was detected in the duodenum (Figure 1A) and jejunum (Figure 1B) of aroused animals but was below the detectable limit in these tissues of torpid animals. TNF-α could not be detected in the large intestine of aroused or torpid animals (Figure 1D). Taken together, the pro-inflammatory cytokines examined in this study were detected in the ileum without significant change between the arousal and torpor states, while more IL-6 and TNF-α were present in the jejunum of aroused versus torpid animals.

### Regulation of anti-inflammatory cytokine protein levels in the intestine during torpor

**Figure 2:**
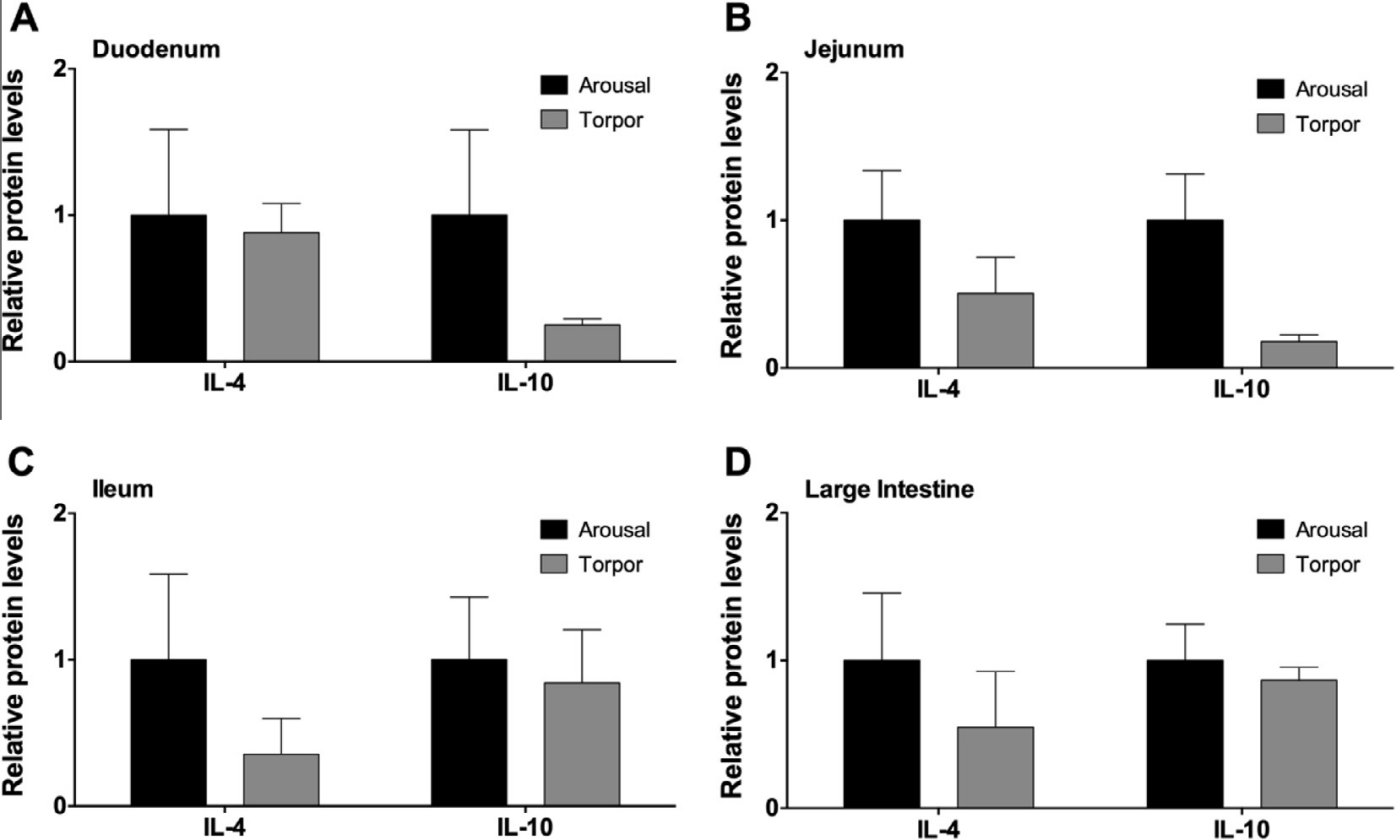
**Relative protein levels of anti-inflammatory cytokines in the intestine of aroused and torpid lemurs** IL-4 and IL-10 protein levels were measured in the duodenum (**A**), jejunum (**B**), ileum (**C**), and large intestine (**D**) using the median fluorescent intensity (MFI) as measured by Luminex®. Data are mean ± SEM (*n* = 3–4) where the values for arousal groups are normalized to a relative value of 1 and torpid groups are expressed as a relative fold change to arousal values. Statistical analysis was performed using a Student’s two-tailed *t*-test, assuming unequal variances.

Production of anti-inflammatory cytokines, such as IL-4 and IL-10, are important in dampening and resolving an inflammatory response [10]. To determine whether an anti-inflammatory response occurred in the gastrointestinal tract of torpid lemurs, the relative protein levels of IL-4 and IL-10 were measured. IL-4 and IL-10 were detected in the duodenum, jejunum, ileum, and large intestine for both aroused and torpid animals (Figure 2). However, there were no significant differences between aroused and torpid animals (*P* > 0.05). Although, IL-10 protein levels appeared to be lower in the duodenum (Figure 2A) and jejunum (Figure 2B) of torpor animals, the high variability of IL-10 levels in aroused animals precluded the ability to detect significant differences.

### Regulation of IL-12 and M-CSF cytokine protein levels in the intestine during torpor

**Figure 3:**
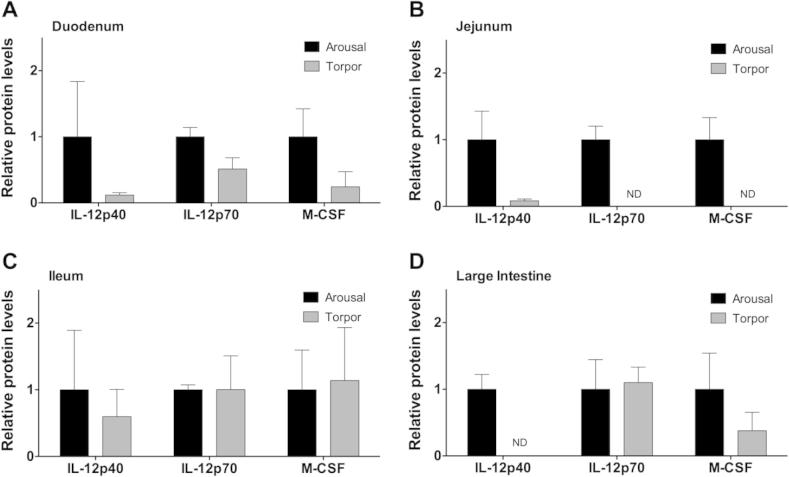
**Relative protein levels of IL-12 subunits and M-CSF in the intestine of aroused and torpid lemurs** IL-12p40, IL-12p70, and M-CSF protein levels were measured in the duodenum (**A**), jejunum (**B**), ileum (**C**), and large intestine (**D**) using the median fluorescent intensity (MFI) as measured by Luminex®. Data are mean ± SEM (*n* = 3–4) where the values for arousal groups are normalized to a relative value of 1 and torpid groups are expressed as a relative fold change to arousal values. Statistical analysis was performed using a Student’s two-tailed *t*-test, assuming unequal variances. ND, not detected, indicating that cytokine protein levels were below the detection limit of the assay.

The pro-inflammatory IL-12p70 cytokine is comprised of two subunits: IL-12p35 and IL-12p40 [9]. While the IL-12p70 heterodimer acts as a pro-inflammatory signal, the IL-12p40 subunit on its own blocks the pro-inflammatory action of IL-12p70 by acting as an antagonist. The IL-12p40 homodimer binds to the IL-12 receptor thereby blocking IL-12p70 mediated intracellular signaling [9]. IL-12p40 was detectable in the duodenum, jejunum, and ileum (Figure 3**A–C**), but there was no significant difference between aroused and torpid animals. In the large intestine, IL-12p40 was detectable in aroused, but not torpid animals (Figure 3D). Protein levels of IL-12p70 were not significantly different between aroused and torpid animals in the duodenum, the ileum, or the large intestine (Figure 3). However, IL-12p70 protein levels were below the detection limit in the jejunum of torpid animals (Figure 3B).

M-CSF has a multitude of activities and is best known for its role in the development of mature macrophages from early myeloid progenitor cells [8]. It also has documented roles in initiating inflammatory responses in mature macrophages [8]. No significant differences, however, were observed in M-CSF levels in the duodenum, ileum, or large intestine (Figure 3A, C, and D) between control and torpid animals. However, while M-CSF levels were detectable in the jejunum of aroused animals, the levels of M-CSF were below the detection limit in the jejunum of torpid animals (Figure 3B).

### Regulation of chemokine protein levels in the intestine during torpor

**Figure 4:**
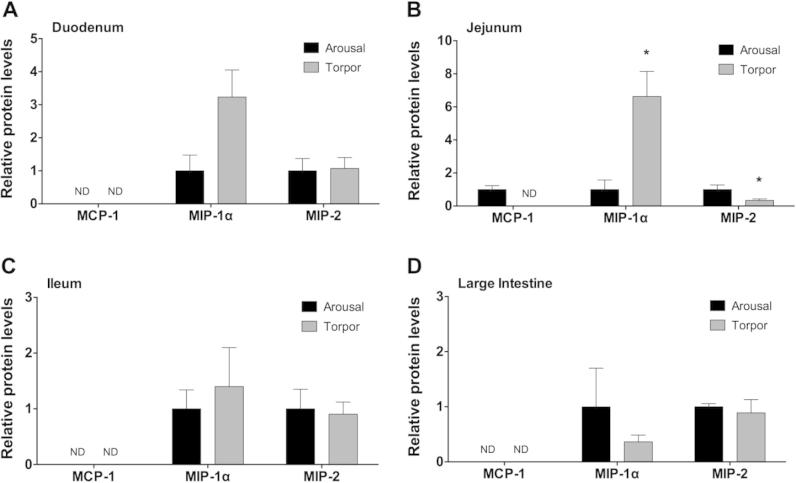
**Relative protein levels of chemokines in the intestine of aroused and torpid lemurs** MCP-1, MIP-1α and MIP-2 protein levels were measured in the duodenum (**A**), jejunum (**B**), ileum (**C**), and large intestine (**D**) using the median fluorescent intensity (MFI) as measured by Luminex®. Data are mean ± SEM (*n* = 3–4) where the values for arousal groups are normalized to a relative value of 1 and torpid groups are expressed as a relative fold change to arousal values. Statistical analysis was performed using a Student’s two-tailed *t*-test, assuming unequal variances. ND, not detected, indicating that cytokine protein levels were below the detection limit of the assay. Asterisks indicate significant differences (*P* < 0.05) in the torpid animals compared to the corresponding aroused animals.

Chemokines are a family of cytokines that act to recruit immune cells to the site of infection, or alternatively, to control cell migration into tissues during homeostasis. To assess the modulation of chemokines in gray mouse lemurs during torpor, the relative levels of the chemokines MCP-1, MIP-1α, and MIP-2 were examined in the different sections of the intestine in aroused and torpid lemurs. MCP-1 was not detected in all intestine samples expect in jejunum of aroused animals (Figure 4). Conversely, MIP-1α and MIP-2 were detected in all samples for both animal states. MIP-1α protein levels were significantly higher in the jejunum of torpid animals compared to those in aroused animals (*P* = 0.029, Figure 4B), however, MIP-1α protein levels did not change significantly in duodenum, ileum or large intestine (Figure 4A, C, and D). Similarly, levels of MIP-2 were comparable between the two states in the duodenum, ileum, and large intestine. However, significantly lower levels of MIP-2 were found in the jejunum of torpid animals compared to those of aroused animals (*P* = 0.049, Figure 4B).

### Regulation of antioxidant defenses in the intestine during torpor

**Figure 5:**
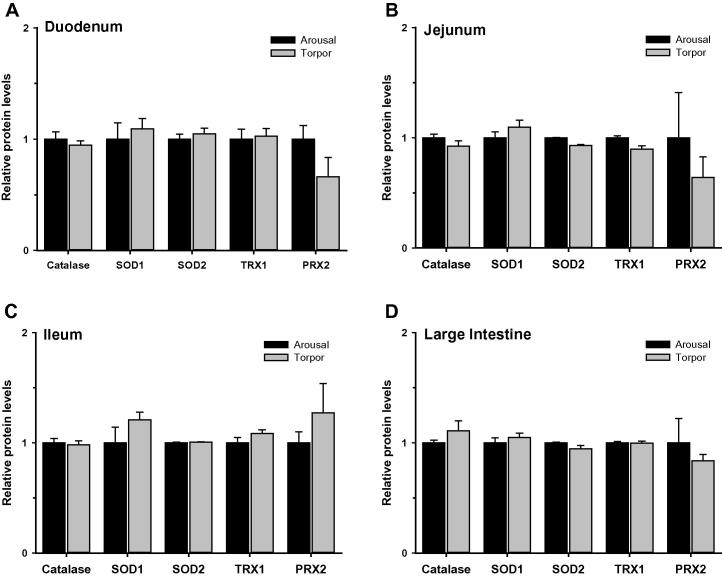
**Relative protein levels of antioxidant enzymes in the intestine of aroused and torpid lemurs** Catalase, SOD1, SOD2, TRX1, and PRX2 protein levels were measured in the duodenum (**A**), jejunum (**B**), ileum (**C**), and large intestine (**D**) using the median fluorescent intensity (MFI) as measured by Luminex®. Data are mean ± SEM (*n* = 3–4) and are expressed relative to arousal values. Statistical analysis was performed using a Student’s two-tailed *t*-test, assuming unequal variances. SOD1, superoxide dismutase 1; SOD2, superoxide dismutase 2; TRX1, thioredoxin 1; PRX2, peroxiredoxin 2.

**Figure 6:**
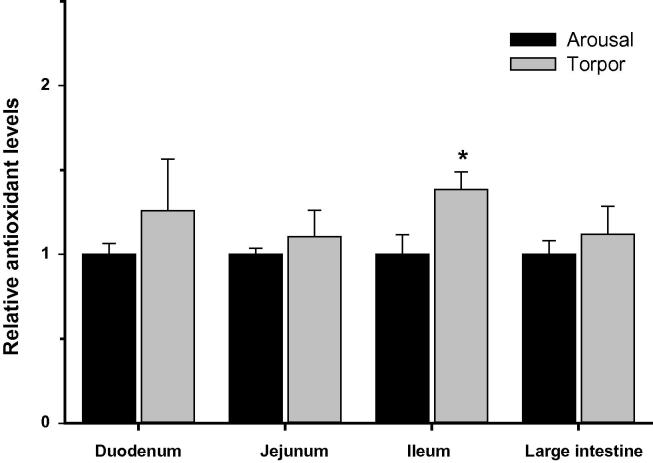
**Relative antioxidant capacity in the intestine of aroused and torpid lemurs** The summed antioxidant capacity provided by low molecular mass metabolites including glutathione, ascorbate (vitamin C), vitamin E, bilirubin, BSA, and uric acid was measured in the duodenum, jejunum, ileum, and large intestine of aroused and torpid lemurs. Mean ± SEM are shown (*n* = 3–4). Data were analyzed using a Student’s two-tailed *t*-test, assuming unequal variances. Significant difference (*P* < 0.05) from the aroused animals is denoted by (^∗^).

Given the link between the immune response and oxidative stress (*e.g.*, respiratory burst), we measured the relative levels of antioxidant enzymes (Figure 5) and metabolites (Figure 6) in the duodenum, jejunum, ileum, and large intestine of aroused and torpid lemurs. First, we examined the protein expression of antioxidant enzymes including catalase, SOD1, SOD2, TRX1 and PRX2. Our results indicated comparable protein levels for all antioxidant enzymes between aroused and torpid lemurs in all the intestinal subsections examined (Figure 5). We then assayed the total antioxidant capacity using the sum of glutathione, ascorbate (vitamin C), vitamin E, bilirubin, bovine serum albumin (BSA), and uric acid as a readout (each are important compliments to the enzymatic antioxidant response). The total antioxidant metabolite capacity in the ileum increased by 40% during torpor, compared to aroused animals (*P* < 0.05) (Figure 6). However, no significant changes were observed in other sections of the intestine between aroused and torpid lemurs.

## Discussion

Daily torpor cycles represent large changes in an animal’s physiology over short periods of time. Along with cessation of feeding, torpor is characterized by lowered *T*_b_ and metabolic rate [15] as well as reduced physiological functions including heart beat and breathing rate. Data collected from free-ranging gray mouse lemurs show that daily torpor bouts are several hours in duration with a minimum T_b_ of ∼27 °C whereas hibernation bouts in this species can last up to 4 weeks with a minimum *T*_b_ of 11.5 °C [16]. Previous studies on torpor and the immune system have shown that immune function can be compromised or depressed during torpor while being restored or, in some cases, heightened during arousal [6]. Studies on little brown bats have demonstrated the susceptibility of this species to white nose disease during hibernation, suggesting a depressed or unresponsive systemic immune response [17–23]. A similar immune suppression response has been seen in the 13-lined ground squirrel, which can enter torpor for a period of 6–40 days [6,24–27]. Hibernating squirrels displayed lower numbers of circulating leukocytes and proliferative splenic T-cells and were unable to respond to injection with lipopolysaccharide (LPS) [25–27]. Indeed, one proposal for the function of interbout arousals in hibernators is that they serve to initiate or restore optimal immune system function for the purposes of dealing with potential pathogenic threats that may be encountered during hibernation [26]. Given that immune system function and oxidative stress pathways are intrinsically linked (*e.g.*, phagocytic leukocytes produce superoxide (O_2_^−^) [11]), we endeavored to characterize their response in the intestine by comparing aroused and torpid gray mouse lemurs.

The intestinal mucosal system is in constant contact with the external environment and is exposed to a high antigenic burden, composed of both commensal and pathogenic microflora [28–33]. As such, the intestinal mucosa is equipped with a tailored immune system comprised of specialized mucosal-associated structures, such as Peyer’s patches, mesenteric lymph nodes, and diffuse immune cells [28–33]. Commensal microbial flora aid in nutrient acquisition and provide metabolic competition with pathogenic microbes. Commensal microbes also help with the development and stimulation of the intestinal immune system, thereby enhancing the capability of the system to fend off pathogenic bacteria [29]. Meanwhile, the general tolerance of the intestinal immune system, in part dictated by regulatory T-cells and the secretion of anti-inflammatory cytokines such as IL-4, IL-10, and TGF-β permits the survival and growth of intestinal microfauna [28,29,32]. Thus, a balance exists between the intestinal immune system and the microbial fauna that exist and persist in the gastrointestinal tract.

Studies examining the effects of torpor and/or seasonal hibernation on the mucosal immune system are limited. Work with 13-lined ground squirrels demonstrated the regulation of intestinal leukocyte numbers and maturation stage, as well as cytokine levels during hibernation [6]. In the present study, examination of pro-inflammatory cytokines, anti-inflammatory cytokines, and chemokines in the duodenum, jejunum, ileum, and large intestine in aroused and torpid animals revealed that the most obvious modulation of cytokine and chemokine levels was in the jejunum. In the jejunum of torpid lemurs, protein levels of the pro-inflammatory cytokines IL-6, TNF-α, IL-12p70, and M-CSF were greatly reduced, often to below the detectable limit of the multiplex cytokine kit. However, protein levels of anti-inflammatory cytokines such as IL-4 and IL-10 in torpid animals were not significantly different from those in aroused lemurs. These results are suggestive of a suppression of the mucosal immune response during torpor and are similar to findings of immune suppression during torpor in hibernators, such as bats and ground squirrels, from cold climates [25]. In the large intestine, higher levels of IL-12p40 were observed in aroused lemurs compared to torpid lemurs, suggesting that IL-12p40 may be produced as a mechanism to limit inflammatory signals mediated by the IL-12 receptor during arousal in this region of the intestine. However, the generalized reduction in levels of pro-inflammatory cytokines may be due to a decrease in the energy budget of the animal during torpor or may, in part, be due to the interruption of feeding and the lowered antigenic burden on the gut during this period [34]. In mammalian species that do not undergo periods of torpor or hibernation, the circadian clock has been shown to govern the systemic immune system as reviewed in [35]. During periods of rest in these mammals, the immune system is also at rest and is activated immediately before the mammals re-enter their active state [35]. Indeed, pathogen challenge experiments have demonstrated that non-hibernating mammals are most susceptible to infection at the end of their rest state, prior to the activation of the immune response [35]. Daily bouts of torpor in the gray mouse lemur may very well involve this same circadian-clock controlled immune system response. However, further studies are warranted to determine if daily torpor exaggerates the effects observed as a result of circadian rhythms. Regardless, the decrease in pro-inflammatory cytokines in the intestinal mucosa of lemurs during torpor may signal an increased risk of infection in the same way as hibernating species that may be underprepared to respond to pathogens during their prolonged torpor bouts [21,22,26,27].

Chemokines play an important role in the immune response since their production is often triggered by a danger signal and acts to recruit varying types of cells to the site of insult. In this study, we examined the protein levels of MCP-1, MIP-1α, and MIP-2. Like the response of the pro-inflammatory cytokines, protein levels of the chemokines, MCP-1 and MIP-2, decreased in the jejunum of torpid lemurs. However, the protein level of MIP-1α was ∼5-fold higher in the jejunum of torpid lemurs and also tended to be higher in the duodenum of torpid lemurs. MIP-1α is chemoattractive to a variety of lymphocytes and leukocytes, but is particularly potent to neutrophils [36]. The increased levels of MIP-1α in the jejunum during torpor may suggest the recruitment of lymphocytes and neutrophils to the intestine tissues. This is in agreement with the decrease in numbers of circulating lymphocytes and neutrophils concurrent with an influx of leukocytes into the gut, which has been observed during periods of torpor in a number of hibernating mammals [6,37] and as reviewed in [22]. Thus, MIP-1α may mediate an influx of neutrophils and lymphocytes into the gut of torpid animals. However, further studies are needed to validate the recruitment of neutrophils into the gut tissues of the gray mouse lemur.

Cycles of torpor and arousal mimic ischemia–reperfusion. During torpor, heart rate and organ perfusion rates are reduced but rise again very rapidly when non-shivering thermogenesis by brown adipose tissue is activated to restore *T*_b_ to a euthermic state. Enhanced cytoprotective strategies (*e.g.*, antioxidant response and unfolded protein response,) are known to play an important role in mammalian hibernators [12,38], suggesting that they may also be important during daily torpor. However, the protein levels of antioxidant enzymes presently measured in the duodenum, jejunum, ileum, and large intestine of aroused versus torpid lemurs were not significantly different. Furthermore, the antioxidant capacity represented by low molecular mass metabolites only increased in the ileum during torpor (by 40%). These data may suggest that the intestinal antioxidant defenses are only minimally required during the relatively shallow torpor experienced by gray mouse lemurs. Indeed, studies of arousal in hibernators indicate that intestinal blood flow is disproportionately low compared to other organs and that the gut is one of the last organs to have normal blood flow restored [14]. Alternatively, antioxidant enzymes of other animals have recently been shown to be regulated by post-translational modifications [39], opening up the possibility that other regulatory mechanisms (apart from changes in protein expression) might modulate antioxidant enzyme responses during primate torpor. Additionally, it is possible that other cytoprotective strategies not presently measured could also play an important role in the gut during primate torpor.

The data herein suggests that the intestinal immune response is suppressed during torpor relative to the aroused state, or conversely, that the immune response is activated in aroused animals relative to the torpor state. As such, gray mouse lemurs may be more vulnerable to pathogen attack via the gut during periods of hypometabolism if the immune system has a reduced ability to control gut pathogens during torpor, potentially allowing for pathogen outgrowth and invasion. However, the antioxidant defense system appears to play a minimal role in the intestine during primate torpor and arousal, although other cytoprotective mechanisms may be important. Finally, future studies may include deeper transcriptomic and/or proteomic analysis on daily torpor to identify more candidate genes, pathways or networks, which may mediate the transitions between daily torpor and arousal.

## Materials and methods

### Animals

Adult female gray mouse lemurs (2–3 years of age, *n* = 8; mean body mass 106.3 ± 15.5 g) used in this study were born in the authorized breeding colony at the National Museum of Natural History (Brunoy, France). Animal holding, experimentation and sampling were conducted by Dr. Martine Perret and the MECADEV team (Mecanismes Adaptatifs et Evolution, Department of Ecology and Management of Biodiversity) as described previously [40,41]. Briefly, mouse lemurs were separated into two experimental groups (*n* = 4 aroused and *n* = 4 torpid). All lemurs were individually housed in cages in a climate chamber, maintained under short day conditions, and torpor–arousal state was assessed by monitoring *T*_b_ and locomotor activity. Animals used for the torpor group were given a calorie-restricted diet for 5 days (60% of the control diet; 86 × 10^−3^ J/day versus a normal diet of 144 × 10^−3^ J/day) to enhance the depth of daily torpor, whereas control animals were fed *ad libitum*. Aroused animals were sacrificed at the end of a daily torpor bout (*T*_b_ 35–36 °C), while torpid lemurs were sacrificed during a torpor bout (when *T*_b_ was at its minimum, 30–33 °C). Animals were euthanized by approved protocols used by the French team (decapitation) and the tissue samples were rapidly excised and immediately frozen in liquid nitrogen. Frozen samples were packed in dry ice and air freighted to Carleton University (Ottawa, Canada) where they were stored at −80 °C until use. The highest standards in ethics and transparency that are applied in Europe were used for all experiments, especially the Directive 86/609/EEC on the protection of animals used for experimental and other scientific purposes.

### Protein homogenates

Protein extracts of frozen duodenum, jejunum, ileum, and large intestine (∼50 mg) were prepared as per the manufacturers’ instructions provided with the Milliplex kits (EMD Millipore, Billerica, MA, USA). Briefly, tissue samples were Dounce homogenized 1:4 w:v in ice-cold lysis buffer (Millipore; catalog No. 43-045) with the phosphatase inhibitors (1 mM Na_3_VO_4_ and 10 mM ß-glycerophosphate) and protease inhibitors (BioShop, Canada, catalog No. PIC001). After incubation on ice with occasional agitation for 30 min, samples were centrifuged at 12,000 × *g* for 20 min at 4 °C and the supernatants were collected as total soluble protein lysates. Soluble protein concentrations were determined using the Bradford assay (catalog No. 500-0005) and then aliquots of lysates were standardized to either 6 or 10 μg/μl for the antioxidant and cytokine panels, respectively. Separate aliquots were used for the antioxidant metabolite assay and were not standardized. Proteins extracts were stored at −80 °C until further use.

### Cytokine multiplex analysis

A customized mouse cytokine/chemokine Milliplex Map kit was ordered from Millipore for analyzing cytokines/chemokines, including IL-1β, IL-4, IL-6, IL-10, IL-12p40, IL-12p70, MCP-1, MIP-1α, MIP-2, and TNF-α. Aliquots of standardized protein lysates (25 μl, 10 μg/μl) were added to individual wells of the supplied 96-well plate. The negative control was 25 μl of lysis buffer (background control). An additional 25 μl of assay buffer was then added to experimental and negative control wells. Mouse cytokine/chemokine quality control 1 (QC1) and quality control 2 (QC2) (catalog No. MXM6070) were used as positive controls; each vial of lyophilized reagent was reconstituted with 250 μl deionized water, vortexed and allowed to sit for 5 min before 25 μl aliquots were added to the positive control wells as per manufacturer’s instructions. All wells then received a further 25 μl of lysis buffer followed by 25 μl of mixed beads. After overnight incubation at 4 °C with continuous orbital shaking, the plate was washed two times with wash buffer. After washing, 25 μl of detection antibodies were then added to each well and incubated for 1 h at room temperature with orbital shaking. Subsequently, 25 μl of streptavidin–phycoerythrin solution was added to each well and incubated for an additional 30 min at room temperature with orbital shaking. Wells were washed two times with 1× wash buffer, re-suspended in 150 μl of sheath buffer, and read on a Luminex® 200 machine with Milliplex Analyst software (Millipore, Billerica, MA).

### Oxidative stress multiplex analysis

The oxidative stress magnetic bead panel (catalog No. H0XSTMAG-18K, Millipore) was used for the analysis of catalase, PRX2, SOD1, SOD2, and TRX1. Tissue extracts were prepared as above, standardized to 6 μg/μl and then aliquots were combined with Milliplex MAP Assay Buffer 1 (catalog No. 43-045) to yield a final working concentration of 0.75 μg/μl, from which 25 μl (18.75 μg total protein) was added to individual wells. The negative control consisted of 25 μl of assay buffer (background control). For the positive control, 25 μl of the provided HepG2 Cell Lysate (unstimulated), prepared as per manufacturer’s instructions, was added to wells. Aliquots of 25 μl of mixed beads were then added to all wells. The plate was incubated for 2 h at room temperature with continuous orbital shaking. Following incubation, the plate was washed three times with 1 × wash buffer. After washing, 50 μl of detection antibodies were added to each well and incubated for 1 h at room temperature with orbital shaking. Wells were once again washed three times with 1 × wash buffer. Subsequently, 50 μl of streptavidin–phycoerythrin solution was added and the plate was incubated for an additional 30 min at room temperature with orbital shaking. Wells were washed three times with 1 × wash buffer, and then magnetic beads were re-suspended in 100 μl of sheath buffer and read on a Luminex® 200 machine with Milliplex Analyst software (Millipore, Billerica, MA).

### Antioxidant capacity assay

Total antioxidant capacity of lemur tissues was evaluated using the antioxidant assay kit from Cayman Chemical Company (catalog No. 709001, Ann Arbor, MI, USA), which measures cumulative antioxidant capacity provided by glutathione, ascorbate (vitamin C), vitamin E, bilirubin, BSA, and uric acid. The assay relies on the ability of antioxidants in the sample to inhibit metmyoglobin-catalyzed oxidation of 2,2′-Azino-di-[3-ethylbenzthiazoline sulfonate] (ABTS). The assay was prepared as per manufacturer’s instructions. Briefly, the standard curve was prepared by combining 10 μl of a Trolox preparation (increasing amounts of reconstituted Trolox diluted with antioxidant assay buffer), 10 μl of metmyoglobin (lyophilized powder reconstituted in antioxidant assay buffer), and 150 μl of Chromogen (containing ABTS reconstituted in HPLC-grade water) per well. Tissue lysates were prepared as described above. Sample wells were prepared by adding 10 μl of a 100-fold dilution of each sample (diluted in antioxidant assay buffer), 10 μl of metmyoglobin, and 150 μl of chromogen. The reactions were initiated by adding 40 μl of 441 μM hydrogen peroxide followed by incubating on a shaker for 5 min at room temperature. Absorbance was read at 750 nm, using a microplate reader, and quantified as Trolox equivalents (mM/mg wet mass).

### Statistical analysis

All numerical data are expressed as means ± SEM (*n* = 4). Statistical analysis was performed using SigmaPlot statistical package (v.12) software. All statistical testing used a two-tailed Student’s *t*-test. Differences were considered significant at *P* < 0.05.

### Authors’ contributions

All authors contributed to the conception and design of the project and to the editing of the manuscript. MP and FP carried out the animal experiments and SNT and BAK conducted biochemical assays. Data analysis and writing of the manuscript were carried out by SNT, BAK, and KBS. All authors read and approved the final manuscript.

### Competing interests

The authors have declared no competing interests.
